# Trends and risk factors of antimicrobial resistance of *Neisseria gonorrhoeae* in Catalonia, Spain, 2022–2023

**DOI:** 10.1007/s10096-026-05456-x

**Published:** 2026-03-28

**Authors:** Mercè Herrero, Sonia Broner, Naiana Pastrana-Batalla, Eduard Anfruns, Evelin López-Corbeto, Victoria González, Judit Serra-Pladevall, Nieves Larrosa, Jacobo Mendioroz, Pilar Ciruela, the Working Group of MRSC, Yuliya Poliakova, Mateu Espasa, Cristina Pitart, Berta Fidalgo, Isabel Pujol, Laura Solaz, Teresa Falgueras, Mayuli Armas, Ester Sanfeliu, Percy Juan Ayala, José Stavola, Carmen Mora, Frederic Gómez, Ester Picó, Araceli González, Claudia Miralles, José Carlos de la Fuente, Eduardo Padilla, Manuel Monsonís, Ferran Navarro, Alba Rivera, Elisenda Miró, Gloria Trujillo, Joan López, Antonio Casabella, Emma Padilla, Lorena Forqué, Sílvia Capilla, Montserrat Olsina, Pepa Pérez, Mar Olga Pérez, Francesc Xavier Queralt, Ester Clapés, Cristina Muñoz, Xavier Clivillé, Alba Bellés, Eric López, Inés Valle, Miriam Campos, Maria Navarro, Marc Garreta, Jesús Trejo, Belén Viñado, Ester del Barrio, Yannick Alan Hoyos, Jordi Cámara, David Sánchez, Rosa Costa, Maria Ángeles Dominguez, Jordi Niubó, Maria Dolores Quesada, Jun Hao Wang Wang, Aida Ramirez Marinero, Núria Torrellas, Rosalía Santos, Olga Gonzalez-Moreno, Albert Barragan, Ariadna Hernández, Elisabet Folch, Geraldine Quelis, Anna Llimós Fabregas, Natàlia Roca, Lourdes Montsant, Miquel Fantova Navarro, Marta Albaiges, Carles Alonso-Tarrés, Carla Benjumea, Manel Panisello, Marta Polo

**Affiliations:** 1 Agència de Salut Pública de Catalunya, Subdirectorate General for Public Health Surveillance and Emergency Response, Barcelona, Spain; 2 Centre d’Estudis Epidemiològics Sobre Les Infeccions de Transmissió Sexual I La Sida de Catalunya (CEEISCAT), Badalona, Spain; 3 Centro de Investigación Biomédica en Red de Epidemiología y Salud Pública (CIBERESP), Instituto de Salud Carlos III, Madrid, Spain; 4 Microbiology Department, Multidisciplinary Inflammation Research Group, Hospital Universitari de Vic, Vic, Spain; 5 Fundació Institut de Recerca I Innovació en Ciències de La Vida I de La Salut de La Catalunya Central, Vic, Spain; 6 Microbiology Department, Vall d’Hebron Hospital Universitari, Barcelona, Spain; 7 Centro de Investigación Biomédica en Red de Enfermedades Infecciosas (CIBERINFEC), Instituto de Salud Carlos III, Madrid, Spain

**Keywords:** *Neisseria gonorrhoeae*, Antimicrobial resistance, Surveillance, Azithromycin, Risk factors, Sexually transmitted infections

## Abstract

**Background:**

*Neisseria gonorrhoeae* (NG) remains a major cause of sexually transmitted infections (STIs) in Europe. Increasing antimicrobial resistance (AMR) threatens treatments’ effectiveness and complicates control efforts. Continuous regional surveillance is essential to detect emerging trends and inform public health action.

**Aim:**

To describe NG epidemiology and AMR trends in Catalonia during 2022–2023, and identify associated resistance risk factors.

**Methods:**

Retrospective analysis of laboratory-confirmed NG cases reported to the Microbiological Reporting System of Catalonia and the STI Registry. Antimicrobial susceptibility testing (AST) was performed for seven antibiotics. Descriptive analysis and logistic regression were used to assess associations between AMR and demographic and clinical variables, including age, sex, sexual orientation and gender identity (SOGI) and anatomical site of infection.

**Results:**

23,968 NG cases were reported, representing an 18.9% increase in incidence from 2022 to 2023. Culture was performed in 5,158 cases, AST results were available for 4,709 isolates. Resistance was highest for ciprofloxacin (71.4%) and tetracycline (48.9%), followed by azithromycin (20.3%) and penicillin (10.2%). Cefixime and ceftriaxone resistance was negligible. Azithromycin resistance was independently associated with homosexual orientation (adjusted odds ratio (aOR): 2.08) and anal infection (aOR: 1.41). Age ≥ 25 years was associated with resistance to ciprofloxacin, tetracycline, and penicillin.

**Conclusions:**

High NG incidence and substantial resistance to older antibiotics, together with notable azithromycin resistance, underscore the need for sustained AMR surveillance, particularly among men who have sex with men (MSM) and in anal infections. Strengthening culture-based AMR surveillance, extragenital screening and targeted prevention strategies are essential to preserve effective treatment options.

## Introduction

*Neisseria gonorrhoeae* (NG) causes gonorrhoea, the second most frequently occurring bacterial sexually transmitted infection (STI) after *Chlamydia trachomatis*, which accounts for the highest number of reported cases worldwide [1]. NG typically colonizes and infects the genital tract, but can also be found in other anatomical sites such as the rectum, oropharyngeal mucosa or conjunctiva [2].

Without treatment, NG can cause serious complications, including pelvic inflammatory disease, that increases the risk of ectopic pregnancies, infertility, miscarriage, fetal death, and congenital infection [1]. Furthermore, NG infection facilitates the acquisition of other STIs and perpetuates transmission in asymptomatic cases [3].

The World Health Organization (WHO) estimated 82 million cases of gonorrhoea worldwide in 2020 [4]. In the European Economic Area (EU/EEA), the incidence has increased by more than 300% between 2008 (29,434 cases) and 2019 (117,985 cases), with countries such as the United Kingdom, Spain, the Netherlands, France, and Sweden accounting for the largest number of cases [5].

The populations at highest risk for NG disease are men who have sex with men (MSM), migrants, the younger individuals (≤ 35 years), and sex workers [3].

NG can develop resistance mechanisms to the antibiotics recommended for its treatment. First- and second-line therapies included sulfonamides, penicillins, tetracyclines, fluoroquinolones, macrolides such as erythromycin, and extended-spectrum cephalosporins (ESC) [6]. However, their use ceased at the end of the last century due to the emergence of antimicrobial resistance (AMR). Since then, newer generation macrolides like azithromycin and third-generation ESC, especially ceftriaxone and cefixime, have become the mainstay treatment, although isolates resistant to these antibiotics have also been reported since the early 2000s and early 2010s [7].

Over the past ten years, in Europe, NG resistance rates to macrolides, tetracyclines, and quinolones have increased, but not to ceftriaxone, which remains below 0.1% − 0.2% [8].

The latest report from the European Gonococcal Antimicrobial Surveillance (Euro-GASP), in which EU/EEA Member States participate [9], concluded that between 2019 and 2020, the proportion of azithromycin-resistant isolates remained constant at 11.0% and 10.1%, respectively. Regarding cefixime, a continuous decreasing trend in resistance levels was observed in 2020 with 0.5% compared to 0.9% in 2019 and 1.4% in 2018. Finally, in both 2018 and 2019, 0.1% of isolates were resistant to ceftriaxone.

In Catalonia, since 2010, the incidence rate of NG has increased by an average of 31.4% annually, from 6.5 in 2010 to 140.4 cases per 100,000 inhabitants in 2022 [10]. Regarding age distribution, the highest number of infections is concentrated in the 25–34 age group, and between genders, males have a ratio of 4:1 over females. During the 2022–2023 period, NG resistance to ciprofloxacin was observed in 71.9% of strains, followed by tetracycline (53.2%), azithromycin (17.4%), and penicillin (10.7%); and, to a lesser extent, 0.15% to cefixime, 0.03% to ceftriaxone, and 0.33% to spectinomycin [11].

Microbiological diagnosis is made by detection of Gram-negative diplococci in stained smears by microscopy, culture, or molecular detection from genital and extragenital samples [11].

The main purpose of the study was to describe and analyze AMR trends of NG during 2022–2023 in Catalonia. The study also aimed to analyze the risk factors associated with antibiotic resistance.

## Methods

### Study design and population

A retrospective cohort study was conducted using data from the Microbiological Reporting System of Catalonia (MRSC) and the Catalan STI Registry based on information collected through an epidemiological survey [12] from January 2022 to December 2023. Our study included both symptomatic and asymptomatic patients, with samples obtained through passive testing and active screening of high-risk populations, including MSM where routine extragenital testing is performed. All laboratory-confirmed cases of NG infection were included using microbiological criteria: isolation of NG in culture, detection of NG nucleic acids (nucleic acid amplification tests, NAAT), or visualization of intracellular Gram-negative diplococci in urethral smears from symptomatic males.

### Data collection

Characteristics of age, sex, anatomical site of infection, and diagnostic methods (culture, Gram stain, or NAAT) were extracted from the MRSC records, and sexual orientation and gender identity from the epidemiological survey.

The site of infection was classified as urethra/balanopreputial/semen, endocervix/vagina, anal canal, pharynx, urine and other (including placenta, blood, joint and peritoneal fluid, biopsy (bone and lymph node), and conjunctiva). For patients with multi-site infections or multiple specimens, only one isolate per patient was included in the analysis. When multiple sites were sampled (e.g., urethral and anal), a single representative isolate was selected according to a predefined hierarchy prioritizing symptomatic sites. Patients contributing multiple tests over time were similarly represented by their first available isolate. For regression analyses, urine isolates were included in “other” to allow inclusion in logistic regression models. Age groups were classified as: ≤ 14, 15–24, 25–34, 35–44, 45–54 and ≥ 55 years. For sub-analyses, age was also dichotomized as < 25 years and ≥ 25 years. The self-reported sexual orientation and gender identity (SOGI) variable was retained according to the original classification collected in the epidemiological survey [12], which jointly gathers information on sexual orientation and gender identity, with categories including heterosexual, homosexual, bisexual, transsexual, and unknown. For analysis purposes, only heterosexual and homosexual were included, without differentiating by sex.

### Antimicrobial susceptibility testing (AST)

Culture and antimicrobial susceptibility testing of NG were performed according to the SEIMC guidelines [13]. A total of 84.2% of laboratories performed Etest or agar dilution, whereas the remaining laboratories used other methods. AST was performed for azithromycin, cefixime, ceftriaxone, ciprofloxacin, penicillin, spectinomycin, and tetracycline. Susceptibility was interpreted according to the European Committee on Antimicrobial Susceptibility Testing (EUCAST) clinical breakpoints (versions 12.0–13.1, 2022–2023) [14, 15]. In EUCAST 2023, tetracycline breakpoints were revised by removing the intermediate category, which reclassified isolates with Minimum Inhibitory Concentration (MIC) ≥ 0.5 mg/L as resistant [15].

### Statistical analysis

Descriptive analyses included Incidence Rate (IR) per 100,000 inhabitants, based on census data from the Statistical Institute of Catalonia. Differences in proportions were assessed using the Chi-square test or Fisher’s exact test, as appropriate.

A comparative analysis of AMR across the periods 2022–2023, 2020–2021, and 2019 was performed. A sub-analysis restricted to individuals who were identified as homosexual or heterosexual was conducted to explore associations between AMR and epidemiological and clinical variables (age, sex, SOGI, and anatomical site of infection). Crude and adjusted odds ratio (OR and aOR) with 95% confidence intervals (CI) were calculated using logistic regression models. A p-value < 0.05 was considered statistically significant.

Statistical analyses were performed using the Statistical Package for the Social Sciences (SPSS) version 24 (IBM Corp., Armonk, NY, USA).

## Results

### Baseline patient characteristics

**Fig. 1:**
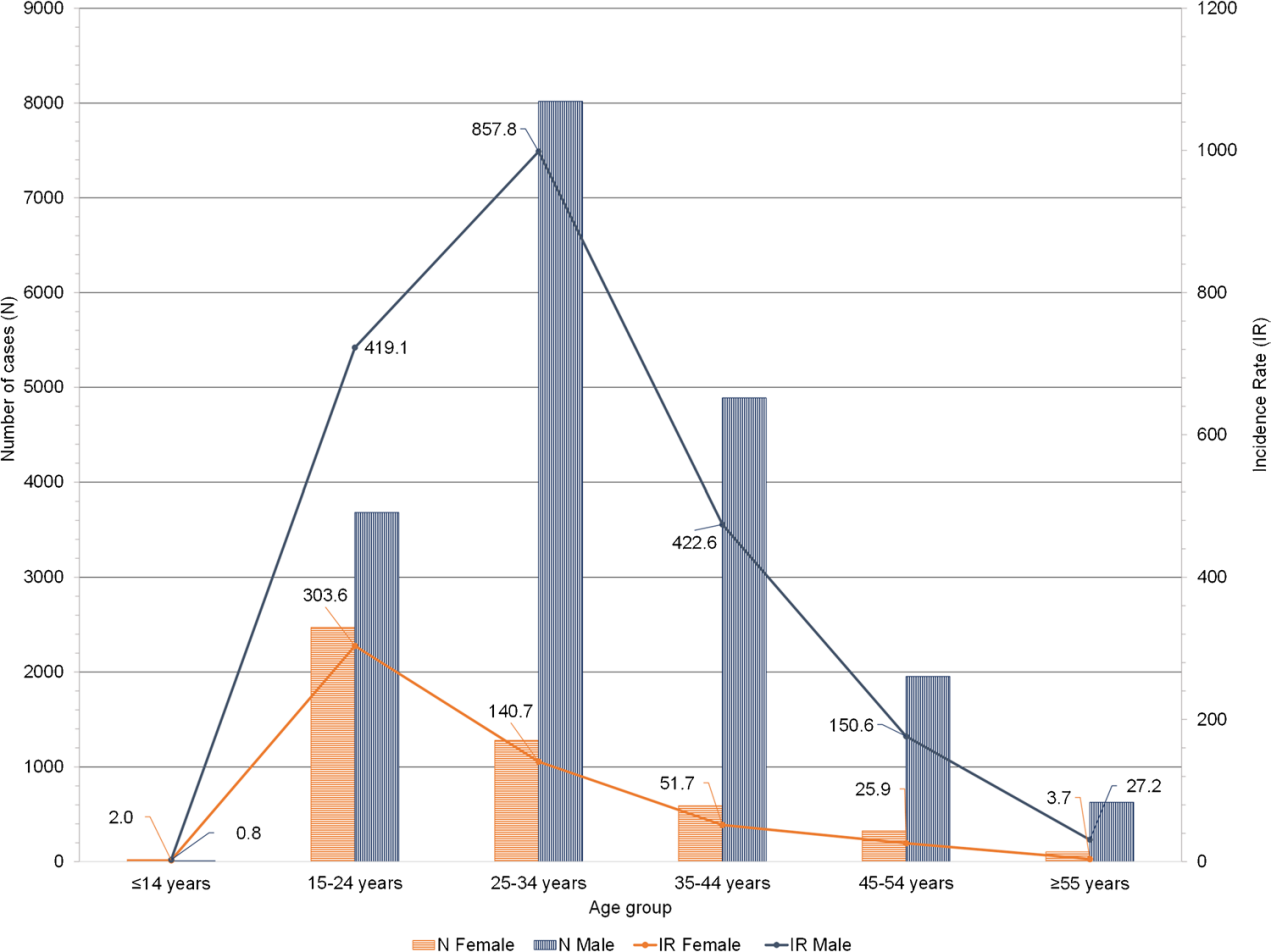
Number and incidence rate of *Neisseria gonorrhoeae* confirmed cases per 100,000 inhabitants by sex and age groups. Catalonia, Spain, 2022–2023

During the 2022–2023 period, 23,968 laboratory-confirmed NG cases were reported, representing an 18.9% increase in the NG incidence rate, from 139.4 per 100,000 inhabitants (10,865 cases) in 2022 to 165.8 cases per 100,000 inhabitants (13,103 cases) in 2023. The IR was markedly higher among males than females (248.4 vs. 60.1/100,000-per year; p < 0.001), except in the pediatric group (≤ 14 years). The median age was 24 years for females (range 2–83 years) and 32 years (0–88) for males. The highest IR was observed in men aged 25–34 years (857.8/100,000 per year), and in women aged 15–24 years (303.6/100,000 per year) (Table 1, Fig. 1).

**Table 1:** Demographic and clinical characteristics of confirmed *Neisseria gonorrhoeae* cases by sex. Catalonia, Spain, 2022–2023

	Female		Male		Total	
	N	%	N	%	N	p-value
Cases	4,789	20.0	19,179	80.0	23,968	< 0.001
IR (per 100,000 inhabitants)	60.1	NA	248.4	NA	165.8	< 0.001
Age in years						
≤ 14 years	22	71.0	9	29.0	31	0.002
15–24 years	2,471	40.2	3,683	59.8	6,154	< 0.001
25–34 years	1,278	13.7	8,018	86.3	9,296	< 0.001
35–44 years	590	10.8	4,888	89.2	5,478	< 0.001
45–54 years	325	14.3	1,954	85.7	2,279	< 0.001
≥ 55 years	103	14.1	627	85.9	730	< 0.001
Median Age (range)	24 (2–83)	NA	32 (0–88)	NA	31 (0–88)	< 0.001
SOGI						
Bisexual	289	42.1	398	57.9	687	< 0.001
Heterosexual	4,019	76.2	1,256	23.8	5,275	< 0.001
Homosexual	93	1.1	8,465	98.9	8,558	< 0.001
Transsexual	45	44.1	57	55.9	102	0.12
Unknown	343	3.7	9,003	96.3	9,346	< 0.001
Sample location						
Urethra/balanopreputial/semen	68	1.1	6,242	98.9	6,310	< 0.001
Anal canal	112	2.7	4,108	97.3	4,220	< 0.001
Endocervix/vagina	3,427	99.7	9^a^	0.3	3,436	< 0.001
Pharynx	1,010	13.5	6,471	86.5	7,481	< 0.001
Urine	108	4.8	2,143	95.2	2,251	< 0.001
Other^b^	64	23.7	206	76.3	270	< 0.001

Abbreviations: IR (per 100,000): incidence rate (per 100,000 inhabitants); N: Number of cases; SOGI: Self-reported sexual orientation and gender identity

^a^Samples correspond to transgender individuals

^b^Other includes: placenta (n = 1), blood (n = 3), joint and peritoneal fluid (n = 50), biopsy (bone and lymph node) (n = 16), conjunctiva in newborns (n = 2), and unknown (n = 198)

SOGI was reported in 61.0% of cases (14,622 cases). Among these, homosexual individuals represented the most affected group at 58.5% of reported individuals, with a strong male predominance (98.9%). Heterosexual individuals accounted for 36.1% of cases, the majority of whom were female (76.2%). Bisexual individuals accounted for 4.7% of cases, with a male predominance (57.9%). No statistically significant differences were observed for transsexual individuals.

Analysis by sample location revealed that pharyngeal samples (n = 7,481; 31.2%) followed by urethral/balanopreputial/semen samples were the most frequently positive (n = 6,309; 26.3%), followed by endocervical/vaginal (n = 4,220 samples; 17.6%), anal canal (n = 3,438; 14.3%) and urine samples (n = 2,250; 9.4%). Only a small number of samples (n = 270; 1.1%) were classified under "other”. Differences between males and females were statistically significant for all sites. NG infections in males were predominantly located in the pharyngeal tract (37.5%), urethral tract (32.5%), anal canal (21.4%), and urine samples (11.2%). However, in females, NG was isolated primarily from the endocervical samples (71.6%).

Regarding diagnostic technique, during the study period (2022–2023), culture was performed in 5,158 cases (21.5%). A 6.4% reduction was observed in 2023 (20.9%) compared to 2022 (22.3%) (p = 0.008). The remaining cases were diagnosed by molecular techniques. Significant differences were found according to the diagnostic method across age groups, sex, and sample location. A higher proportion of NG cases diagnosed by culture was observed in individuals aged 15–24 years (28.0%) and those older than 55 years (4.3%), whereas cases in the 25–44-year age group (23.3% and 39.5%, respectively) were more frequently diagnosed by PCR. Men were predominantly diagnosed by culture (86.9%), while women were more often diagnosed by PCR (21.9%). Regarding specimen type, culture was the most frequently used diagnostic method for urethral/semen/balanopreputial samples (66.3%). AST results were available for 4,709 isolates (91.3%). Among the NG cases that included antibiogram information, the most frequently analyzed antibiotic was ciprofloxacin (4,639 cases; 98.5%), followed by penicillin (4,469 cases; 94.9%), azithromycin (4,177 cases; 88.7%), ceftriaxone (3,667 cases; 77.9%), cefixime (3,334 cases; 68.7%), tetracycline (2,832 cases; 60.1%), and spectinomycin (919 cases; 19.5%) (Table 2).

**Table 2:** Antibiotic resistance among *Neisseria gonorrhoeae* isolates in Catalonia, Spain, 2019 vs. 2020–2021 and 2022–2023

	2019			2020–2021			2020–2021 vs. 2019		2022–2023			2022–2023 vs. 2019	
	Total	Resistant		Total	Resistant		DIF%	p-value	Total	Resistant		DIF%	p-value
Antimicrobial	N	N	%	N	N	%			N	N	%		
Ciprofloxacin	1896	1170	61.7	2222	1404	63.2	2.4	0.3	4639	3336	71.9	16.5	< 0.001
Tetracyclin	762	249	32.7	923	311	33.7	3.1	0.7	2832	1295	45.7	39.9	< 0.001
Azithromycin	1662	273	16.4	1946	301	15.5	−5.8	0.5	4177	726	17.4	5.8	0.397
Penicillin	1854	389	21.0	2153	537	24.9	18.9	< 0.001	4469	477	10.7	−49.1	< 0.001
Cefixime	1166	22	1.9	1368	3	0.2	−88.4	< 0.001	3334	5	0.1	−92.1	< 0.001
Spectinomycin	436	3	0,7	334	5	1,5	117,6	0,5	919	3	0,3	−52,6	0,618
Ceftriaxone	1608	15	0,9	1841	17	0,9	−1,0	0,9	3667	1	0,0	−97,1	< 0,001

Abbreviations: DIF%: relative difference in resistance percentage between 2019 and 2020–2021 and 2022–2023; N: number of tested isolates

^a^Resistance for azithromycin defined by the EUCAST epidemiological cut-off (ECOFF) value (MIC > 1 mg/L, 2019 [14, 15])

Penicillin resistance increased by 18.9%, from 21.0% in 2019 to 24.9% in 2020–2021 (p < 0.001), whereas it decreased by 49.1%, from 21.0% in 2019 to 10.7% in 2022–2023 (p < 0.001). Ciprofloxacin resistance increased by 16.5%, from 61.7% in 2019 to 71.9% in 2022–2023 (p < 0.001), and tetracycline resistance increased by 39.9%, from 32.7% to 45.7% (p < 0.001). Cefixime resistance decreased by 88.4%, from 1.9% in 2019 to 0.2% in 2020–2021 (p < 0.001), and by 92.1%, from 1.9% in 2019 to 0.15% in 2022–2023 (p < 0.001) (Table 2).

### Antimicrobial resistance patterns

**Fig. 2:**
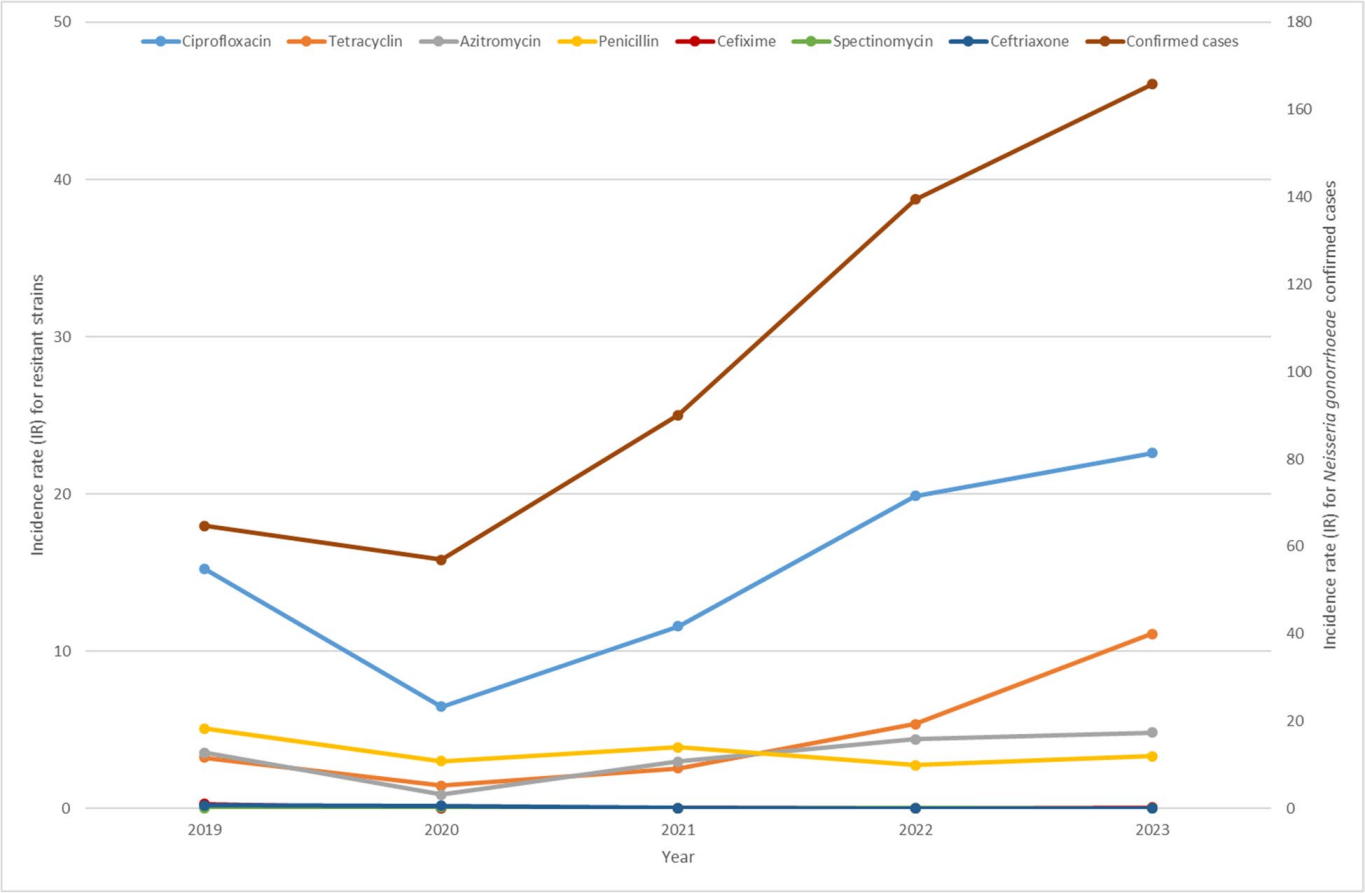
Incidence rate of *Neisseria gonorrhoeae* confirmed cases and incidence rate of resistant strains per 100,000 inhabitants. Catalonia, Spain, 2019–2023

The NG incidence rate during the 2022–2023 period increased by 236% and 137% compared with 2020–2021 and 2019, respectively. Increased incidence rates were also observed for all antibiotic resistance studied, particularly for ciprofloxacin, tetracycline, and azithromycin (Fig. 2).

Antimicrobial susceptibility patterns of NG isolates differed slightly depending on the SOGI of the individuals from whom the samples were obtained. A sub-analysis was performed that included only individuals identified as homosexual or heterosexual due to their prevalence in the dataset and the sample size being sufficient for statistical comparison (Table 3).

**Table 3:** Distribution of antimicrobial resistant *Neisseria gonorrhoeae* isolates by SOGI in homosexual and heterosexual population. Catalonia, Spain, 2022–2023

	Heterosexual		Homosexual		Total	
Antibiotic	N	%^a^	N	%^a^	N	p-value
Spectinomycin	2	100.0	0	0.0	2	0.317
Ciprofloxacin	601	38.0	979	62.0	1580	< 0.001
Penicillin	86	39.6	131	60.4	217	< 0.001
Tetracycline	211	38.9	332	61.1	543	< 0.001
Azithromycin	105	24.9	301	71.3	422	< 0.001
Cefixime	1	50.0	1	50.0	2	1.000

Abbreviations: N: number of tested isolates, SOGI: Self-reported sexual orientation and gender identity

^a^Percentages were calculated by row (by antibiotic)

All isolates tested for ceftriaxone were completely susceptible, with no resistance detected in either heterosexual or homosexual individuals.

Similarly, resistance to spectinomycin was exceptionally low and not significant, with only two resistant isolates (0.07%) identified in heterosexual individuals and none in homosexual individuals. For cefixime, only two isolates (0.07%) were resistant (one in each group).

In contrast, resistance to azithromycin was significantly more prevalent in isolates from homosexual individuals (71.3%) compared to those from heterosexual individuals (24.9%) (p < 0.001). A similar pattern was observed for the other antimicrobials analyzed. In the case of ciprofloxacin, resistance rates of 62.0% were detected in homosexual individuals and 38.0% in heterosexual individuals (p < 0.001). For tetracycline, resistant strains were also higher in homosexual individuals (61.1%) than in heterosexual individuals (38.9%) (p < 0.001). Finally, in the case of penicillin, 60.4% of homosexual individuals were resistant to NG compared with 39.6% in heterosexual individuals (p < 0.001).

Association between patient characteristics and antimicrobial resistance in *Neisseria gonorrhoeae.*

In homosexual and heterosexual individuals, univariate and multivariate analyses were performed to assess the association between key variables such as age (< 25 years, ≥ 25 years), sex, SOGI, site of infection and resistance to four antibiotics: azithromycin, ciprofloxacin, penicillin, and tetracycline in NG isolates, which accumulate to an appropriate number of resistant isolates (Table 4).

**Table 4:** Association of antimicrobial resistance of *Neisseria gonorrhoeae* and epidemiological and clinical variables in the homosexual and heterosexual population. Catalonia, Spain, 2022–2023

		Total isolates tested N		Resistant isolates N		Resistant isolates %	OR (95%CI)		aOR (95%CI)	
**Azithromycin**										
Total		2001		406		20.3				
**Age, years**										
< 25		476		71		14.9	1		1	
≥ 25		1525		335		22.0	**1.61 (1.21–2.12)**		1.26 (0.93–1.71)	
**Sex**										
Female		415		71		17.1	1		1	
Male		1586		335		21.1	1.3 (0.98–1.72)		0.51 (0.24–1.1)	
**SOGI**										
Heterosexual		737		105		14.2	1		1	
Homosexual		1264		301		23.8	**1.88 (1.47–2.4)**		**2.08 (1.41–3.07)**	
**Site of infection**										
Urethra/Balanopreputial/semen		983		182		18.5	1		1	
Anal canal		304		86		28.3	**1.74 (1.29–2.34)**		**1.41 (1.04–1.92)**	
Endocervix/vagina		366		60		16.4	0.86 (0.63–1.19)		0.81 (0.38–1.73)	
Pharynx		310		72		23.2	1.33 (0.98–1.81)		1.11 (0.81–1.53)	
Others		38^a^		6		15.8	0.83 (0.34–2)		0.95 (0.38–2.35)	
**Ciprofloxacin**										
Total		2212		1580		71.4				
**Age, years**										
< 25		544		367		67.5	1		1	
≥ 25		1668		1213		72.7	**1.29 (1.04–1.59)**		**1.3 (1.03–1.64)**	
**Sex**										
Female		478		340		71.1	1		1	
Male		1734		1240		71.5	1.02 (0.82–1.27)		1.12 (0.6–2.07)	
**SOGI**										
Heterosexual		854		601		70.4	1		1	
Homosexual		1358		979		72.1	1.09 (0.9–1.31)		0.94 (0.71–1.23)	
**Site of infection**										
Urethra/Balanopreputial//semen		1115		774		69.4	1		1	
Anal canal		318		242		76.1	**1.4 (1.05–1.87)**		**1.39 (1.03–1.88)**	
Endocervix/vagina		421		300		71.3	1.09 (0.85–1.4)		1.26 (0.67–2.35)	
Pharynx		318		235		73.9	1.25 (0.94–1.65)		1.26 (0.94–1.68)	
Others		40^b^		29		72.5	1.16 (0.57–2.35)		1.21 (0.59–2.47)	
**Penicillin**										
Total	2137		217		10.2					
**Age, years**										
< 25	520		38		7.3			1		1
≥ 25	1617		179		11.1			**1.58 (1.1–2.27)**		1.25 (0.99–1.59)
**Sex**										
Female	456		42		9.2			1		1
Male	1681		175		10.4			1.15 (0.8–1.63)		1.19 (0.64–2.21)
**SOGI**										
Heterosexual	809		86		10.6			1		1
Homosexual	1328		131		9.9			0.92 (0.69–1.23)		0.93 (0.7–1.23)
**Site of infection**										
Urethra/Balanopreputial//semen	1064		120		11.3			1		1
Anal canal	319		24		7.5			0.64 (0.41–1.01)		**1.43 (1.05–1.93)**
Endocervix/vagina	400		34		8.5			0.73 (0.49–1.09)		1.32 (0.7–2.5)
Pharynx	313		37		11.8			1.06 (0.71–1.56)		1.28 (0.95–1.72)
Others	41^c^		2		4.9			0.4 (0.1–1.69)		1.23 (0.6–2.52)
**Tetracycline**										
Total	1109		543		49.0					
**Age, years**										
< 25	311		112		36.0			1		1
≥ 25	798		431		54.0			**2.09 (1.59–2.73)**		**1.63 (1.21–2.19)**
**Sex**										
Female	280		101		36.1			1		1
Male	827		442		53.4			**2.02 (1.53–2.68)**		0.68 (0.3–1.58)
**SOGI**										
Heterosexual	530		211		39.8			1		1
Homosexual	579		332		57.3			**2.03 (1.6–2.58)**		**1.51 (1.09–2.1)**
**Site of infection**										
Urethra/Balanopreputial//semen	682		364		53.4			1		1
Anal canal	75		42		56.0			1.11 (0.69–1.8)		0.94 (0.58–1.54)
Endocervix/vagina	254		87		34.3			0.46 (0.34–0.61)		0.46 (0.2–1.08)
Pharynx	68		31		45.6			0.73 (0.44–1.21)		0.65 (0.39–1.08)
Others	30^d^		19		63.3			1.51 (0.71–3.22)		1.66 (0.76–3.62)

CI: confidence interval; N: number of tested isolates; aOR: adjusted odds ratio; OR: crude odds ratio; SOGI: Self-reported sexual orientation and gender identity

^a^: Other includes urine (30), joint and peritoneal fluid (1), biopsy (bone and lymph node) (1), and unknown (6)

^b^: Other includes urine (31), joint and peritoneal fluid (2), biopsy (bone and lymph node) (1), and unknown (6)

^c^: Other includes urine (31), joint and peritoneal fluid (2), biopsy (bone and lymph node) (1), and unknown (7)

^d^: Other includes urine (27), and unknown (2)

Of 2,001 isolates, 20.3% (n = 406) were resistant to azithromycin. In univariate analysis, azithromycin-resistant strains were associated with age ≥ 25 years (OR: 1.61; 95%; CI: 1.21–2.12), homosexual orientation (OR: 1.88; 95% CI: 1.47–2.4), and anal canal strains (OR: 1.74; 95% CI: 1.29–2.34). In multivariate analysis, homosexual orientation (aOR: 2.08; 95% CI: 1.41–3.07) and anal canal samples (aOR: 1.41; 95% CI: 1.04–1.92) remained associated with azithromycin resistance.

Regarding ciprofloxacin, of 2,212 isolates analyzed, 71.4% (n = 1,580) presented resistance. Age ≥ 25 years (OR: 1.29; 95% CI: 1.04–1.59) and anal canal samples (OR: 1.40; 95% CI: 1–05-1.87) were associated with ciprofloxacin resistance which was maintained in the multivariate analysis (aOR: 1.3; 95% CI: 1.03–1.64 and aOR: 1.39; 95% CI: 1.03–1.88, respectively).

Of the 2,137 isolates analyzed, 10.2% (n = 217) were resistant to penicillin. Penicillin-resistant strains were associated with age ≥ 25 years (OR 1.58; 95% CI: 1.10–2.27). In the multivariate analysis, an association was observed only with anal canal samples (aOR 1.43; 95% CI: 1.05–1.93).

Nearly half of the samples analyzed (48.9%; n = 543) were tetracycline resistant. In univariate analysis, tetracycline-resistant strains were associated with age ≥ 25 years (OR 2.09; 95%CI: 1.59–2.73), male (OR: 2.02; 95%CI: 1.53–2.68), and homosexual orientation (OR: 2.03; 95% CI: 1.6–2.58). In multivariate analysis, age ≥ 25 years (aOR: 1.63; 95% CI: 1.21–2.19) and homosexual orientation (aOR: 1.51; 95% CI: 1.09–2.1) remained associated.

## Discussion

This study presents microbiological and epidemiological surveillance data from 23,968 confirmed cases of NG in Catalonia over a two-year period. We integrated individual risk profiles with AMR results to assess associations in cases diagnosed in both primary care centres and hospitals.

A notable rise in the incidence of gonococcal infection during 2022–2023 was observed, with an overall IR of 152.7 cases/100,000 inhabitants, and an increase of 236.1% compared to 2019 (IR 64.7/100,000) [16].

This upward trend is also evident in the EU/EEA and Spain. In the EU/EEA, the reporting rate increased by 31% between 2022 and 2023, while between 2014 and 2023, the reporting rate increased by 321%, except in 2020, the first year of the COVID-19 pandemic, when it decreased. In Spain, the reporting rate increased by more than 50% between 2021 and 2023 [17]. Factors such as the strengthening of epidemiological surveillance systems, broad screening policies—including asymptomatic individuals—as recommended by WHO [18], and an increase in risky sexual behaviors such as unprotected sex or having multiple partners, may contribute to the increase in incidence [19].

Not all European countries or other regions of Spain have implemented screening measures with the same intensity as Catalonia. However, the sustained trend of increase in STIs in Catalonia emphasizes the urgent need to reinforce education and prevention strategies [10].

Affected women were younger than men, with a median age of 24 years versus 32 years, respectively. Although other studies have reported similar age differences, they differences were less pronounced. For instance, Williamson et al. noted a median age of 29 years for men and 27 years for women [20].

In Catalonia, young men aged 15–24 years—particularly among MSM—are more likely to present with symptoms and therefore undergo PCR testing at free screening sites, with positive samples subsequently cultured. This testing strategy may partly explain the higher culture rates observed in this group, as well as the distribution of specimen sites. Regional surveillance data indicate that gonorrhoea disproportionately affects young men and MSM in Catalonia, reflecting both underlying epidemiological risk and targeted testing practices [10].

The marked increase in gonorrhoea incidence during 2022–2023 was followed by a concomitant rise in resistance rates, particularly to ciprofloxacin, tetracycline, and azithromycin. This temporal concordance suggests a possible ecological association, whereby higher transmission and case burden may lead to increased antimicrobial exposure and treatment pressure, favouring the selection and dissemination of resistant strains [21, 22]. Similar post-pandemic recrudescence in STI incidence have been reported in Europe (including Spain), raising concerns about their potential impact on AMR dynamics and future treatment effectiveness (European Centre for Disease Prevention and Control (ECDC) [17]. 

Resistance to ciprofloxacin and tetracycline increased significantly in 2022–2023 compared with 2019, aligning with global trends in *N. gonorrhoeae* AMR [23]. In Catalonia, ciprofloxacin resistance rose from 61.7% in 2019 to 71.9% in 2022–2023, and tetracycline resistance from 32.7% to 45.7%. In contrast, resistance to penicillin declined markedly (from 21.0% to 10.7%), and resistance to cefixime and ceftriaxone decreased to near elimination (from 1.9% to 0.1% and from 0.9% to 0.0%, respectively). These trends likely reflect the implementation of dual therapy guidelines in 2020 and strengthened surveillance efforts. They are consistent with national data from Spain and European surveillance (Euro-GASP), which report persistently low resistance to extended-spectrum cephalosporins but variable resistance rates for other antimicrobials. Although WHO surveillance has reported the emergence of ceftriaxone-resistant strains globally, often associated with mosaic *penA* alleles [24], the continued low ceftriaxone resistance observed in Catalonia may reflect region-specific factors such as antimicrobial stewardship practices, population dynamics, and comprehensive extragenital screening. Ongoing genomic and AMR surveillance remains essential to promptly detect the emergence of resistant strains.

No significant changes in spectinomycin resistance were observed (0.7% in 2019 and 0.3% in 2022–23), consistent with its limited clinical use and low global resistance rates. A recent meta-analysis of 432,880 NG isolates revealed that resistance to spectinomycin remains exceptionally low (around 0.3%), with a decreasing trend over time [24].

In addition, azithromycin resistance remained stable in 2022–2023 at 17.4%, while in Europe the level of resistance was highest (25.6%) in 2022 and increased compared to 2021 (14.2%) [9].

Azithromycin remains a major concern due to its prior inclusion in dual therapy for gonorrhoea in previous guidelines. The associations between antimicrobial resistance and MSM status, anal infection, and age ≥ 25 years are likely multifactorial. Evidence suggests that dense sexual networks, repeated exposure to antimicrobials, and frequent screening and treatment in MSM populations may facilitate both selective pressure and clonal dissemination of resistant strains [25]. Differences across antibiotics may reflect heterogeneous historical use and selective pressures, particularly for macrolides and tetracyclines, as well as site-specific dynamics at extragenital reservoirs [8, 26]. In our study, homosexual orientation was independently associated with azithromycin resistance, consistent with other studies and Euro-GASP findings, which also identified higher resistance rates among MSM. In 2022, most azithromycin-resistant isolates were linked to MSM- and anorectal or oropharyngeal infections [27]. These associations may be attributed to repeated antimicrobial exposure, clustered sexual networks, and elevated STI screening frequency in this population [28]. Azithromycin resistance was also associated with anal canal isolates, consistent with previous evidence reporting higher resistance rates in rectal samples [8]. This location is known to present challenges in clearance, and asymptomatic presentation may facilitate persistent and transmissible resistant strains [29].

Resistance to ciprofloxacin was the most prevalent, affecting 71.4% of isolates and was higher than in Europe (65.9%) [9]. This antibiotic was excluded from empirical therapy in international guidelines in 2007 [30]. Associations were observed between ciprofloxacin resistance and both age ≥ 25 years and anal canal isolates. These findings reinforce the importance of including extragenital samples in the STI screening and diagnostic protocols to detect resistant infections that may otherwise be missed [31]. Penicillin resistance was observed in 10.2% of isolates and only anal canal samples showed an association with penicillin resistance. Rectal infections, particularly common among MSM, may facilitate genetic exchange among NG strains, contributing to resistance dissemination. This site-specific resistance may reflect local microbial ecology or differential antibiotic tissue penetration, as previously proposed [32].

Tetracycline resistance was high (48.9%), consistent with global resistance patterns linked to prolonged use of older antibiotics [23]. In England, NG tetracycline resistance was higher than in our study, increasing from 39.4% to 62.9% between 2016 and 2020. The increased use of doxycycline for STIs and non-STI pathogens may contribute to tetracycline resistance in NG through a bystander effect [33]. Age ≥ 25 years was strongly associated with tetracycline resistance, suggesting a cumulative exposure effect. Although resistance was more common in males in univariate analysis, adjusted models revealed no significant association. Homosexual orientation remained a significant predictor, reinforcing behavioral or network-related transmission dynamics. Unlike other antibiotics, tetracycline resistance did not vary significantly by infection site, suggesting a broad, less localized distribution of resistant strains.

European treatment guidelines recommend dual therapy with high doses of ceftriaxone and azithromycin, or monotherapy with high doses of ceftriaxone, which is now more commonly used. It is important to note that resistance to azithromycin is increasing in different countries, and although the level of resistance to cefixime has decreased significantly, close monitoring of this resistance is necessary. Simply increasing the doses of ESC will likely be ineffective in the long term, so some authors recommend continuing dual therapy and complementing it with enhanced surveillance for antimicrobial resistance [34].

A key strength of this study is the representativeness of the samples, drawn from all hospitals and care centres across Catalonia over a two-year period. Additionally, suspected cases reported by healthcare professionals were actively investigated, validated by epidemiologists, and confirmed by microbiology laboratories. However, the number of cultures decreased over time due to the increasing use of NAAT-based diagnostics, resulting in a lower-than-expected proportion of cases tested for antimicrobial resistance, as resistance data in our setting are available from culture-confirmed cases.

Furthermore, the SOGI variable was availablez in approximately 60% of cases, and although this information needs to be improved, it is an acceptable percentage for determining the results. The SOGI variable in our dataset reflects the structure of the original epidemiological survey, which combined sexual orientation and gender identity concepts into a single question with predefined, mutually exclusive response categories. This design limited the possibility of capturing non-exclusive combinations (e.g., heterosexual and transgender). In updated versions of the survey, this limitation has been addressed by including separate variables for gender identity and sexual behavior, enabling more detailed and accurate data collection.

Other methodological considerations should be taken into account. The lack of individual-level data on prior antimicrobial use precluded differentiation between primary and acquired resistance and limited the assessment of factors underlying the higher AMR observed among MSM and individuals aged ≥ 25 years. Nevertheless, the observed resistance patterns were consistent with national and global surveillance data, supporting the robustness and generalisability of our findings. The absence of test-of-cure data, particularly for extragenitourinary infections, further precluded direct evaluation of treatment outcomes and their potential influence on resistance trends. AST coverage was high for most antibiotics but lower for spectinomycin, likely reflecting its limited clinical use; this reduced coverage may have limited the precision of resistance estimates. Finally, the inclusion of both symptomatic and screening-detected infections, particularly among MSM, reflects routine surveillance practice and should be considered when comparing resistance estimates with studies restricted to symptomatic cases. Overall, the large sample size and population-based design support the robustness and reliability of our findings, which provide valuable population-level estimates of AMR to inform ongoing surveillance and public health decision-making.

## Conclusion

These results underscore the complex and multifactorial nature of gonococcal resistance. While age and SOGI consistently influenced resistance patterns for some antibiotics, these associations were not uniform across all agents. The consistent finding of higher resistance at extragenital sites such as the anal canal highlights the need for comprehensive anatomical screening, particularly in MSM and other high-risk populations.

Resistance patterns underscore the importance of extragenital screening and targeted interventions for high-risk groups.

Continued AMR surveillance and prevention strategies are essential to control the spread of resistant NG strains. Future public health strategies should focus on strengthening culture-based diagnostic capacity, enhancing partner notification and treatment adherence, and incorporating molecular diagnostics to detect resistance determinants. Tailored interventions that consider behavioral, demographic, and anatomical factors are crucial to limiting further spread of resistant NG and preserving remaining effective treatment options.

Box. Main findings of the study.

**Table:** 

Gonorrhoea incidence in Catalonia increased by 236% from 2019 to 2022–2023, reaching 152.7 cases per 100,000 population
High Resistance Rates for:
Ciprofloxacin: 71.4% resistant—no longer viable for empirical treatment
Tetracycline: 48.9% resistant—consistent with global trends
Azithromycin: 20.3% resistant—very concerning due to past use in dual therapy
Penicillin: 10.2% resistant
These findings reveal a continuous increase in resistance to some antibiotics (tetracycline and ciprofloxacin) historically used for gonorrhoea, even after discontinuation
Risk Factors for Resistance:
**Sex:** Not associated
**Age ≥ 25 years**: Associated with increased resistance to ciprofloxacin, tetracycline, and penicillin
**Homosexual orientation**: Significantly associated with resistance to azithromycin (aOR 2.08) and tetracycline (aOR 1.51). Individuals with homosexual orientation were more likely to be infected with *Neisseria gonorrhoeae* strains resistant to azithromycin and tetracycline, and this link remained strong even when other variables were controlled for
**Extragenital sites:**
Anal canal: Higher resistance to azithromycin, ciprofloxacin, and penicillin

## Data Availability

All the data used in the analysis was collected during routine public health surveillance activities, as part of the legislated mandate of the Health Department of Catalonia, the competent authority for the surveillance of communicable diseases, which is officially authorized to receive, treat and temporarily store personal data on cases of infectious diseases. All data was fully anonymized.
